# Clinical Significance of High-Risk HPV Positivity with Negative Cytology: Can Further Evaluation Be Safely Delayed?

**DOI:** 10.3390/medicina62081493

**Published:** 2026-08-03

**Authors:** Aleksandar R. Živadinović, Aleksandra N. Petrić, Lazar R. Živadinović, Ivan R. Ilić, Dušan D. Simić, Sonja Z. Pop-Trajković Dinić, Milan S. Trenkić, Jelena D. Milošević Stevanović, Nataša K. Rančić

**Affiliations:** 1 Clinic for Gynecology and Obstetrics, University Clinical Center Niš, 18000 Niš, Serbia; zivadinovicaleksandar92@gmail.com (A.R.Ž.); sanja.petric@hotmail.com (A.N.P.); lazarzivadinovicmedfakni@gmail.com (L.R.Ž.); sonjapoptrajkovic@gmail.com (S.Z.P.-T.D.); trenkic@gmail.com (M.S.T.);; 2 Faculty of Medicine, University of Nis, 18000 Niš, Serbia; ilicko81@gmail.com (I.R.I.); dusan.m.simic@gmail.com (D.D.S.); 3 Center for Pathology and Pathological Anatomy, University Clinical Center Niš, 18000 Niš, Serbia; 4 Women’s Health Protection Office, Health Center Niš, 18000 Niš, Serbia; 5 Institute for Public Health Niš, 18000 Niš, Serbia

**Keywords:** high-risk *human papillomavirus*, cervical cancer screening, cytology triage, p16/Ki-67 dual-stain biomarkers, CIN2+ lesions

## Abstract

*Background and Objectives*: Primary HPV screening has high sensitivity in detecting premalignant and malignant cervical lesions. However, due to its relatively low specificity, this screening approach requires triage tests. Owing to its high specificity, cytology is most commonly employed in the triage of HPV-positive results. Nevertheless, the question remains as to how reliable cytology is in triaging these cases, given its relatively low sensitivity. This study evaluates the clinical significance of high-risk HPV (hrHPV) positivity in women with negative or low-grade cytological findings and assesses whether further evaluation can be safely deferred using HPV genotyping, colposcopy, and p16/Ki-67 biomarkers. *Materials and Methods*: A prospective hospital-based observational study was conducted on 125 women with a positive hrHPV cervical test. All participants underwent conventional cytology, extended HPV genotyping, colposcopy, and histopathological evaluation via biopsy or loop excision. Immunohistochemical analysis of p16 and Ki-67 expressions was performed on histopathological tissue samples. Particular attention was focused on women with negative for intraepithelial lesion or malignancy (NILM) and low-grade cytological findings. Associations between cytology, HPV genotype distribution, colposcopic findings, histopathology, smoking status, and biomarker expression were analyzed. *Results*: Among HPV-positive patients with NILM cytology, CIN2+ lesions were histopathologically confirmed in 28.0% of cases. More than half (51.4%) of all CIN2+ lesions detected in HPV-positive women were identified in patients with low-grade or negative cytology. HPV16/18 positivity was highly prevalent among women with CIN2+ lesions and low-grade cytological findings (78.4%). Unsatisfactory colposcopy with a non-visible squamocolumnar junction (transformation zone type 3, TZ3) was identified in more than half of these patients. Immunohistochemical markers p16 and Ki-67 demonstrated high sensitivity (95.5%) and negative predictive value (93.3%) for detecting CIN2+ lesions in hrHPV-positive women with low-grade cytology (NILM, ASC-US, and LSIL). Smoking status was significantly associated with CIN2+ lesions in this patient group (*p* < 0.001). *Conclusions*: Deferring further evaluation of hrHPV-positive women solely based on negative cytology may be unsafe, particularly in older women, smokers, and those positive for HPV16/18. The strong association between p16/Ki-67 positivity and CIN2+ lesions suggests that dual-stain testing directly from cytological samples could significantly improve triage strategies and reduce the need for invasive procedures.

## 1. Introduction

With an estimated 604,000 new cases and 280,000 deaths worldwide in 2024, cervical cancer (CC) remains the fifth most commonly diagnosed cancer and the fourth leading cause of cancer death among women [1]. *Human papillomavirus* (HPV) infection is a necessary but insufficient cause of CC, with 13 of 118 known HPV types classified as group one carcinogens by International Agency for Research on Cancer (IARC) Monographs [2]. Other important cofactors include sexually transmitted infections, smoking, high parity, immunosuppressive drug use (e.g., transplantation recipients), and low intake of certain micronutrients (including carotenoids and vitamin E) [3]. Its unique natural history, well-established causal association with persistent infection by high-risk hrHPV, recognizable precancerous lesions, and the availability of effective primary and secondary prevention strategies make CC a model disease for cancer prevention. Well-organized screening programs and prophylactic HPV vaccination have substantially reduced the incidence and mortality of CC in many high-income countries. Nevertheless, the disease continues to impose a considerable burden, particularly in settings where screening coverage remains inadequate or preventive strategies have not been fully implemented [4,5,6].

Despite decades of cytology-based screening, CC has not been eliminated, even in high-income countries. A substantial proportion of invasive cancers occur in women who have never participated in screening, have been inadequately followed after abnormal findings, or have false-negative screening results. Furthermore, cytology has inherent diagnostic limitations, particularly in detecting lesions located high within the endocervical canal, glandular precancerous lesions, adenocarcinoma in situ, and invasive adenocarcinoma, where cytological and colposcopic examinations may demonstrate reduced diagnostic accuracy [7,8]. Recognizing hrHPV as the necessary etiological agent for cervical carcinogenesis has fundamentally transformed CC prevention. Molecular HPV testing detects high-risk viral genotypes with substantially greater sensitivity than cytology for identifying cervical intraepithelial neoplasia of grade 2 or worse (CIN2+) and grade 3 or worse (CIN3+). Evidence from large randomized controlled trials and pooled analyses has consistently demonstrated that primary HPV-based screening provides significantly greater protection against invasive CC than cytology alone while allowing for longer screening intervals following a negative HPV test [6,7,8]. Consequently, HPV testing has become the preferred primary screening strategy in many countries owing to its superior clinical performance and favorable cost-effectiveness [9]. Accordingly, several countries, including Australia, the United Kingdom, the Netherlands, Sweden, Denmark, and Turkey, have replaced or are progressively replacing cytology with primary HPV testing in their national cervical cancer screening programs [10]. In contrast, conventional cytology remains the primary screening method in Serbia. National cervical cancer screening guidelines introduced in 2013 recommend cytological screening every three years for women aged 25–64 years [11]. However, population-based data indicate that participation in regular screening remains suboptimal, with considerably fewer women being screened within the recommended interval than expected. Pilot implementation studies evaluating the feasibility of primary HPV screening are currently underway, highlighting the need for evidence that may support future modifications to the national screening strategy [12,13].

Although primary HPV screening has substantially improved the detection of cervical precancer, it has also created new challenges in the clinical management of women who test positive for hrHPV but have negative cytology (hrHPV-positive/NILM). This group represents approximately 30–40% of all HPV-positive women identified through population-based screening programs [12,13,14]. While primary HPV testing has excellent sensitivity for detecting CIN2+ lesions, its specificity is lower than that of cytology, particularly among younger women, in whom transient HPV infections are common. Most HPV infections are spontaneously cleared within one to two years, and only a small proportion of hrHPV-positive women will develop high-grade cervical lesions or invasive CC [15,16]. Consequently, managing women with hrHPV-positive/NILM results has become one of the most important unresolved issues in CC screening. Immediate referral of all HPV-positive women to colposcopy would result in unnecessary diagnostic procedures, increased healthcare costs, patient anxiety, and potential overtreatment. Conversely, delaying diagnostic evaluation in women with persistent hrHPV infection may increase the risk of missing clinically significant precancerous lesions. Therefore, accurate risk stratification is essential to identify women who require immediate colposcopic assessment while safely allowing those at low risk to continue surveillance [17,18].

Current evidence indicates that the risk associated with a positive HPV test is not uniform but depends on several factors, including HPV genotype, persistence of infection, previous screening history, and individual risk profile. Longitudinal studies have consistently shown that women with persistent hrHPV infection have a substantially higher risk of developing CIN3+ than women with newly detected infections following previous negative HPV tests [19,20]. Among individual genotypes, HPV16 carries the highest absolute risk of CIN3+, whereas HPV18 is strongly associated with adenocarcinoma in situ and invasive adenocarcinoma of the cervix. Other oncogenic genotypes, such as HPV31 and HPV33, are associated with intermediate risk, whereas HPV56 has one of the lowest risks among high-risk HPV types [21,22,23]. To improve clinical decision-making, several triage strategies have been incorporated into international screening guidelines. HPV16/18 genotyping is currently recommended by numerous professional organizations, including the American Society for Colposcopy and Cervical Pathology (ASCCP), to identify women requiring immediate colposcopy. Women infected with other hrHPV genotypes are generally managed according to cytological findings and individualized risk estimates [24]. However, HPV genotyping alone does not identify all women at risk for clinically significant disease. Consequently, additional triage approaches, including dual-stain p16/Ki-67 cytology, extended HPV genotyping, DNA methylation biomarkers, and artificial intelligence-assisted cytology, are being investigated to improve the specificity of primary HPV screening while maintaining its high sensitivity [25,26,27,28,29]. Although international risk-based management guidelines provide evidence-based recommendations for women with hrHPV-positive/NILM results, most available data originate from countries where HPV testing has been established as the primary screening method for many years. Evidence from countries where conventional cytology remains the primary screening test, including Serbia, is still limited. Differences in screening organization, participation rates, HPV genotype distribution, and healthcare resources may influence the applicability of international recommendations to local populations.

Epidemiological data from Serbia indicate that CC continues to represent a substantial public health burden, while participation in organized screening remains below the desired level [30]. Furthermore, the national CC screening program in Serbia is currently based on cytology, whereas HPV testing is being introduced gradually. Therefore, it is essential to establish triage algorithms that are feasible within the real-world setting of the national healthcare system. Such algorithms should consider the availability of extended HPV genotyping, colposcopy, histopathological examination, and immunohistochemical analyses, as well as the fact that p16/Ki-67 dual-stain testing on cytology specimens is not yet routinely available across all healthcare facilities. Consequently, evaluating the combined diagnostic value of HPV genotyping, cytology, colposcopy, and p16/Ki-67 biomarkers is of particular clinical and practical importance. In this context, improving the triage of HPV-positive women could facilitate more efficient use of healthcare resources, enable earlier detection of clinically significant cervical lesions, and reduce unnecessary diagnostic and therapeutic procedures. Particular attention should be paid to identifying women in whom negative cytology should not be considered sufficient justification for postponing further diagnostic evaluation.

The following hypotheses were tested:

**H1.**  *Negative cytology in an HPV-positive woman should not be considered sufficient evidence to safely defer further diagnostic evaluation.*

Our findings further support this statement, as a substantial proportion of women with histologically confirmed CIN2+ lesions had negative or low-grade cytological findings. This indicates that relying solely on cytology may result in missed clinically significant lesions and delayed diagnosis. Therefore, negative cytology in HPV-positive women should not be considered sufficient justification for postponing colposcopic evaluation or other appropriate diagnostic procedures, particularly in the presence of additional risk factors or high-risk HPV genotypes.

**H2.**  *A proportion of patients with low-grade cytological findings have histopathologically confirmed CIN2+ lesions.*

A subset of women with low-grade cytological findings was found to have histopathologically confirmed CIN2+ lesions.

**H3.**  *Conventional cytology is equally reliable as an independent triage method for HPV-positive women.*

We agree that conventional cytology remains an established and valuable triage method for HPV-positive women. However, its sensitivity for the detection of CIN2+ lesions is not optimal, and clinically significant lesions may still be present despite negative or low-grade cytological findings. In our study, a proportion of HPV-positive women with NILM or low-grade cytological abnormalities had histopathologically confirmed CIN2+ lesions, demonstrating that cytology alone may not identify all women at clinically relevant risk. Therefore, while conventional cytology represents an important component of HPV-positive women management, our findings support the need for a risk-based approach that incorporates additional clinical information, including HPV genotype, colposcopic findings, and other relevant risk factors, particularly in women with persistent HPV infection or other indicators of increased risk.

**H4.**  *HPV16/18 genotyping improves the sensitivity of cytology-based triage.*

The identification of HPV16 and HPV18 infections allows the detection of women at higher risk for CIN2+ lesions, even when cytological abnormalities are absent or of low grade. In our study, the presence of histologically confirmed CIN2+ lesions among women with negative or low-grade cytological findings highlights the limitations of cytology alone as a triage method. Therefore, incorporating HPV16/18 genotyping into the diagnostic algorithm may improve the identification of women requiring closer surveillance or further diagnostic evaluation.

**H5.**  *Dual-stain p16/Ki-67 positivity is associated with CIN2+ detection.*

The simultaneous expression of p16 and Ki-67 reflects cell-cycle deregulation induced by high-risk HPV infection and may improve the identification of women with clinically significant cervical lesions. Although dual-stain testing was not evaluated in our study, our findings support the need for complementary biomarkers and diagnostic approaches beyond cytology alone. In our cohort, CIN2+ lesions were detected among women with negative or low-grade cytological findings, emphasizing the limitations of cytology-based triage and the potential value of additional biomarkers, such as p16/Ki-67 dual staining, in improving the detection of women at increased risk.

**H6.**  *Smoking is associated with the presence of CIN2+ lesions.*

In our study, smoking was significantly associated with the presence of CIN2+ lesions in the univariate analysis. However, this association did not remain statistically significant in the multivariable logistic regression model after adjustment for other potential confounding factors. These findings suggest that, although smoking may be associated with CIN2+ lesions, its independent contribution could not be confirmed in our cohort. The observed association in the univariate analysis may partly reflect the influence of other risk factors associated with cervical intraepithelial neoplasia.

**H7.**  *Regular screening attendance is associated with the detection of CIN2+ lesions.*

Although regular screening attendance was significantly associated with CIN2+ detection in the univariate analysis, this association was not confirmed in the multivariable model.

**H8.**  *Combining cytology, HPV genotyping, and dual-stain testing improves triage performance compared with cytology alone.*

Combining cytology with HPV genotyping and dual-stain testing may improve triage performance compared with cytology alone by increasing the identification of women at risk for CIN2+ lesions. These complementary approaches provide additional risk stratification, particularly among HPV-positive women with negative or low-grade cytological findings. Although HPV genotyping and dual-stain testing were not evaluated in our study, our findings support the need for improved triage strategies beyond cytology alone. In our cohort, a proportion of women with NILM or low-grade cytological abnormalities had histopathologically confirmed CIN2+ lesions, highlighting the limitations of cytology-based triage and the potential benefit of incorporating additional biomarkers and molecular risk assessment tools in future diagnostic algorithms.

## 2. Materials and Methods

### 2.1. Study Design

This prospective hospital-based observational study included 125 women aged 30 to 65 years with a positive high-risk HPV (hrHPV) cervical test. All participants underwent conventional cytology, extended HPV genotyping, colposcopy, and histopathological evaluation by cervical biopsy or loop excision.

### 2.2. Sample

Among all women examined at the specialized outpatient clinic of the University Clinical Center Niš from 1 August 2025, to 1 May 2026, 288 with a positive hrHPV cervical test were identified according to the predefined selection criteria. Based on the inclusion criteria, 125 women were ultimately enrolled. The remaining women—163—were excluded based on predefined criteria.

The inclusion criteria were as follows:

- Aged between 30 and 65 years;
- Positive hrHPV cervical test;
- Positive high-risk HPV test from a cervical sample;
- Extended HPV genotyping performed;
- Cervical cytological examination performed;
- Signed informed consent for participation.

**Figure 1:**
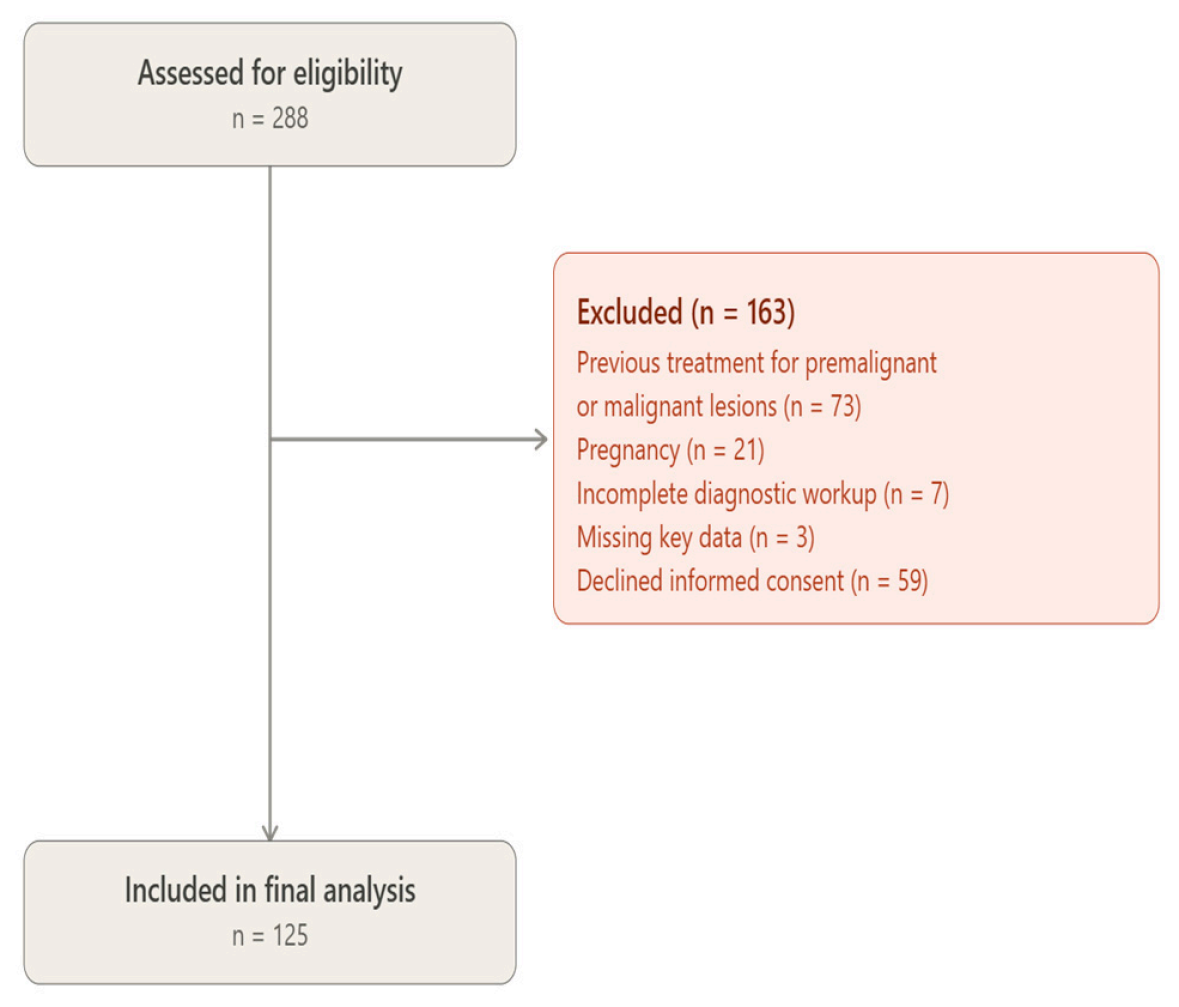
Flowchart of participant selection and inclusion in the final analysis.

The exclusion criteria were as follows:

- Previous treatment for premalignant or malignant cervical lesions;
- Pregnancy;
- Incomplete diagnostic workup, preventing reliable comparison of the investigated methods;
- Missing key data required for statistical analysis;
- Refusal to provide informed consent (Figure 1).

The primary study outcome was the presence of histopathologically CIN2+. CIN2+ was selected as the primary endpoint because it represents clinically significant cervical lesions that require further diagnostic evaluation, treatment, or close surveillance. This allowed us to assess the ability of different diagnostic and triage methods to identify women with lesions that should not be missed during the triage process. Moreover, CIN2+ is widely recognized as a clinically relevant threshold for evaluating the diagnostic performance and clinical utility of cervical cancer screening and triage strategies.

Because histopathological assessment was performed on all participants, the risk of partial verification bias was minimized. However, as this was a hospital-based study, the findings may not be directly generalizable to the general cervical cancer screening population.

### 2.3. Data Collection

We used a structured questionnaire specifically designed for this study. The first section collected socio-demographic characteristics, including age, occupation, marital status, and smoking habits. The second section included questions regarding parity, attendance at routine annual gynecological examinations, and previous ablative procedures.

All participants underwent a comprehensive clinical evaluation, including a gynecological examination, collection of medical history and socioepidemiological data, cervical cytology, extended HPV genotyping, colposcopic examination, and histopathological assessment. Immunohistochemical analysis of p16 and Ki-67 expression was also performed on patients from whom histopathological specimens were obtained.

Immediately after sample collection, cervical specimens, together with the corresponding documentation, were transported to the Institute of Public Health Niš, where HPV testing and extended HPV genotyping were performed. HPV genotyping was carried out using a Multiplex Real-Time Polymerase Chain Reaction (PCR) assay. This method directly detects and amplifies genotype-specific segments of *human papillomavirus* (HPV) DNA, enabling the individual identification of HPV genotypes present in each specimen. The assay provides extended HPV genotyping by individually detecting the following HPV genotypes: HPV16, HPV18, HPV31, HPV33, HPV35, HPV45, HPV51, HPV52, HPV53, HPV56, HPV58, HPV66, HPV68, and HPV69. Therefore, all genotype-specific results should be interpreted within the scope of the HPV panel included in the assay.

Cytological findings were classified according to the Bethesda System [30]. Based on the colposcopic examination, participants were evaluated according to the type of transformation zone, the adequacy of colposcopy (satisfactory or unsatisfactory), and the severity of the colposcopic findings. Colposcopic findings, including transformation zone type and lesion grade, were classified according to the official International Federation for Cervical Pathology and Colposcopy (IFCPC) colposcopic terminology [31].

Histopathological diagnoses were classified as cervicitis, CIN1, CIN2, CIN3, or carcinoma. CIN2+ lesions were considered clinically significant outcomes because they required further surgical treatment.

Immunohistochemical expression of p16 and Ki-67 was evaluated in histopathological specimens to confirm and refine the histopathological diagnosis. Among all evaluated biomarkers, p16 and Ki-67 showed the strongest association with the presence of CIN2+ lesions. Positive expression of these biomarkers was associated with more than 50-fold higher odds of CIN2+ lesions.

Due to technical limitations associated with conventional cytology at our institution, dual-stain analysis could not be performed directly on cytological specimens.

#### Ethical Considerations

This study was conducted in accordance with the Declaration of Helsinki. The study protocol was approved by the Ethics Committee of the University Clinical Center Niš (Decision No. 19408/8, dated 4 July 2025) and by the Ethics Committee of the Primary Health Care Center Niš (Decision No. 1501/9-10, dated 13 May 2025).

All participants received detailed information regarding the study and provided written informed consent before enrollment.

### 2.4. Statistical Analysis

Statistical analysis was performed using IBM SPSS Statistics, version 26 (IBM Corp., Armonk, NY, USA). Continuous variables are presented as the mean ± standard deviation (SD) or as the median with the interquartile range (IQR), depending on the distribution of the data, while categorical variables are expressed as absolute and relative frequencies. Associations between categorical variables were assessed using Pearson’s chi-square test or Fisher’s exact test, as appropriate. The diagnostic performance of p16, Ki-67, and HPV16/18 was evaluated by calculating sensitivity, specificity, positive predictive value (PPV), negative predictive value (NPV), and overall diagnostic accuracy for the detection of CIN2+ lesions. Univariate logistic regression analysis was performed to identify factors associated with the presence of CIN2+ lesions. The results were expressed as odds ratios (ORs) with corresponding 95% confidence intervals (95% CIs). Variables that were statistically significant or considered clinically relevant were included in a multivariable logistic regression model to identify independent predictors of CIN2+ lesions. The results of the multivariable logistic regression analysis were reported as ORs with their corresponding 95% CIs.

All statistical tests were two-sided, and a *p*-value of <0.05 was considered statistically significant.

## 3. Results

A total of 125 women participated in the study, and their mean age was 46.51 ± 13.76 years (range: 30 years to 65 years). Table 1 presents the baseline characteristics of the study participants.

The mean age of the participants was 46.51 ± 13.76 years. When categorized by age groups, the largest proportion of women was aged 36–45 years (31.2%), followed by those aged 46–55 years (30.4%). Women aged 30–35 years accounted for 19.2% of the study population, while 19.2% were aged 56–64 years. Regarding parity, the highest proportion of women had two children (24.0%), followed by those with three children (21.6%), more than four children (20.0%), no children (18.4%), and one child (16.0%). This distribution of reproductive characteristics allowed us to assess potential associations between reproductive factors and the severity of histopathological findings. Most participants reported attending regular annual gynecological examinations (68.8%), whereas 31.2% did not. This finding indicates a discrepancy between self-reported participation in gynecological follow-up and adherence to cytological screening practices, which is relevant for the interpretation of subsequent analyses involving previous screening history. Regarding smoking status, most participants reported smoking one pack of cigarettes per day (56.0%), 8.0% smoked more than one pack daily, and 36.0% were non-smokers. A positive family history of cervical cancer or other relevant gynecological malignancies was reported in approximately one-quarter of participants, while postcoital bleeding was reported by approximately one-fifth of women.

Table 2 presents the clinical, colposcopic, cytological, and histopathological characteristics of the study participants.

Analysis of the clinical, cytological, colposcopic, histopathological, and immunohistochemical characteristics demonstrated that HPV genotypes 16 and/or 18 were the most frequently detected genotypes in the study population. HPV16 was the predominant individual genotype, followed by HPV18 as the second most prevalent. Among the remaining detected genotypes, HPV31, HPV53, and HPV58 were the most common, whereas all other genotypes were identified at lower frequencies. A negative for intraepithelial lesion or malignancy (NILM) cytological result was observed in 28.0% of participants. Low-grade and borderline cytological abnormalities represented the most common cytological category, including atypical squamous cells of undetermined significance (ASC-US), atypical glandular cells not otherwise specified (AGC-NOS), and low-grade squamous intraepithelial lesions (LSIL). Atypical squamous cells, including HSIL (ASC-H), and atypical glandular cells, including neoplasia (AGC-FN), were identified in approximately one-quarter of participants, whereas high-grade cytological abnormalities were detected in a smaller proportion of participants, including high-grade squamous intraepithelial lesions (HSILs), lesions more severe than HSIL, adenocarcinoma in situ (AIS), and lesions more severe than AIS. This distribution indicates that a substantial proportion of HPV-positive women did not present with unequivocal high-grade cytological abnormalities, representing the principal clinical challenge addressed in the present study. Colposcopic examination was unsatisfactory in 43.2% of participants. Grade 1 colposcopic abnormalities were identified in 32.8%, whereas Grade 2 abnormalities were observed in 24.0%. The Type I transformation zone was the most frequently observed, followed by Type III, while Type II was the least common. The high proportion of unsatisfactory colposcopic examinations and Type III transformation zones suggests significant limitations of colposcopy in this population, particularly among women in whom the squamocolumnar junction was partially or completely inaccessible to direct visualization. Histopathological examination revealed cervicitis in 30.4% of participants, cervical intraepithelial neoplasia grade 1 (CIN1) in 16.8%, CIN2 in 12.0%, CIN3 in 20.0%, and invasive cervical carcinoma in 20.8% of women. When CIN2, CIN3, and invasive carcinoma were combined and classified as clinically significant CIN2+ lesions, these lesions were confirmed in 66 of 125 participants (52.8%). This finding indicates a high burden of clinically significant histopathological abnormalities within the study population and further emphasizes the need for accurate risk stratification and triage of HPV-positive women.

Positive p16 expression was detected in 64.0% of participants, while positive Ki-67 expression was observed in an identical proportion (64.0%). These findings indicate a high prevalence of biomarkers associated with transforming HPV infection and increased cellular proliferative activity in the study population.

Table 3 shows the distribution of high-risk HPV genotypes outside the HPV16/18 group according to their oncogenic potential.

Among these, HPV31 and HPV53 were the most frequently detected types, each associated with CIN2+ lesions in 13.6% of cases, followed by HPV58, which was identified in 7.6% of cases.

Table 4 presents the association between cytological findings and the presence of CIN2+ lesions.

The distribution of cytological findings differed significantly between women with and without CIN2+ lesions (*p* = 0.009). Normal cytology (NILM) was more frequently observed among women without CIN2+ lesions than among those diagnosed with CIN2+ (35.6% vs. 21.2%). Conversely, high-grade cytological abnormalities (HSIL/AIS) were substantially more common in women with CIN2+ lesions than in those without CIN2+ lesions (19.7% vs. 1.7%). Notably, 21.2% of women with histologically confirmed CIN2+ lesions had a negative cytological result (NILM), representing false-negative cytology findings. In 34.3% of CIN2+ cases, the cytological diagnosis underestimated lesion severity compared with the final histopathological diagnosis. Overall, 44.9% of women with CIN2+ lesions (24.2% with low-grade abnormalities and 19.7% with high-grade abnormalities) had cytological findings that were consistent with the histopathological diagnosis. In contrast, only 1.7% of women with abnormal cytological findings had a benign histopathological result on biopsy. These findings support the well-established observation that cytology is a highly specific but relatively insensitive diagnostic method for the detection of clinically significant cervical lesions. The greatest diagnostic challenge was associated with equivocal cytological categories between NILM and LSIL and between LSIL and HSIL, including ASC-US, AGC-NOS, ASC-H, and ASC-FN. A proportion of these borderline cytological findings were nearly identical in women with and without CIN2+ lesions (39.1% vs. 34.8%, and 23.7% vs. 24.2%, respectively). One explanation for the high prevalence of equivocal cytological diagnoses is the inherent subjectivity of cytological interpretation. In addition, knowledge of a patient’s HPV-positive status may influence reporting behavior, potentially leading cytologists to avoid classifying findings as entirely normal when subtle abnormalities are present. This phenomenon may contribute to the increased frequency of borderline cytological categories observed in HPV-positive women (Table 4). 

Among women with CIN2+ lesions and low-grade cytological findings (NILM, ASC-US, AGC-NOS, and LSIL), the highest absolute number of cases was observed in the age groups 46–55 years (35.1%) and 36–45 years (32.4%). However, when the proportion of CIN2+ lesions in each age category was analyzed, a progressive increase in CIN2+ frequency with advancing age was observed. The lowest proportion of CIN2+ lesions was recorded among women aged 30–35 years (12.5%), whereas the highest proportion was found in women aged 56–65 years (37.5%), followed by those aged 46–54 years (34.2%) and 36–45 years (30.7%). These findings suggest that clinically significant cervical lesions may be present despite negative or low-grade cytological findings, particularly among older women (Table 5).

Table 6 presents the HPV infection characteristics in women with CIN2+ lesions despite low-grade or negative cytology.

HPV16/18 positivity was highly prevalent in this subgroup, being detected in 78.4% of cases, whereas only 21.6% of women were infected with other high-risk HPV genotypes. Most women had a single-type HPV infection (81.1%), while multiple HPV infections were less common (18.9%). The high prevalence of HPV16/18 among women with normal or low-grade cytology and histologically confirmed CIN2+ lesions suggests that patients harboring these genotypes should undergo colposcopic and histopathological evaluation regardless of cytological findings. In contrast, the presence of multiple HPV genotypes was not significantly associated with the detection of CIN2+ lesions compared with single-type infections. These findings raise the question of whether targeted biopsy should be considered even in women with mild (G1) colposcopic changes when hrHPV positivity is accompanied by negative or low-grade cytology. This consideration may be particularly relevant given that some HSILs can present as thin HSILs originating directly from reserve cells and may not produce pronounced colposcopic abnormalities.

Table 7 presents the distribution of colposcopic findings among women with histologically confirmed CIN2+ lesions according to their cytological findings, comparing patients with negative or low-grade cytology (NILM, ASC-US, AGC-NOS, and LSIL) and those with high-grade cytological abnormalities whose cytological diagnoses corresponded to the histopathological findings.

Colposcopy was unsatisfactory (transformation zone type 3 (TZ3) with a non-visible squamocolumnar junction) in 51.4% of women with negative or low-grade cytological findings and in 57.9% of women with cytological abnormalities corresponding to histologically confirmed CIN2+ lesions. Among women with satisfactory colposcopy (TZ1 or TZ2), colposcopic examination identified abnormal findings in all cases with histologically confirmed CIN2+ lesions. However, among women with false-negative or low-grade cytology, 21.6% demonstrated only grade 1 (G1) colposcopic abnormalities. According to current colposcopic practice, such mild findings would generally not prompt biopsy. Consequently, approximately 21.6% of women with CIN2+ lesions and negative or low-grade cytology could have remained undiagnosed if biopsy had been restricted to women with more severe colposcopic abnormalities. These findings raise the question of whether targeted biopsy should also be considered in hrHPV-positive women with negative cytology who present only with mild (G1) colposcopic changes. This issue may be particularly relevant because some HSILs, including so-called thin HSILs, may arise directly from reserve cells and may not produce prominent colposcopic abnormalities. Unsatisfactory colposcopy (TZ3) remains a particular challenge in managing hrHPV-positive women. In our cohort, CIN2+ lesions were identified in nearly 51% of participants with negative or low-grade cytology and an unsatisfactory colposcopic examination, indicating that clinically significant lesions may remain undetected when evaluation relies solely on cytological findings and visualization of the transformation zone.

Table 8 demonstrates a statistically significant association between p16 and Ki-67 expressions and histopathological diagnosis (*p* < 0.001).

p16 negativity was most frequently observed in cervicitis (81.6%) and decreased progressively with increasing lesion severity, being absent in CIN3 and rare in invasive carcinoma (3.8%). Conversely, p16 positivity increased markedly with disease severity, reaching 100% in CIN3 and 96.2% in carcinoma cases. Similarly, Ki-67 negativity was predominantly found in cervicitis (86.8%) and was uncommon in higher-grade lesions. Ki-67 positivity demonstrated a strong increasing trend across histopathological categories, reaching 93.3% in CIN2, 96.0% in CIN3, and 96.2% in carcinoma. When analyzed according to the presence of clinically significant lesions (Table 9), p16 and Ki-67 demonstrated high diagnostic performance in detecting CIN2+ lesions. Sensitivity was 95.5%, and specificity was 71.2% for both biomarkers. The PPV was 78.8%, while the NPV reached 93.3% for CIN 3 (96 0%) and carcinoma (96.2%).

p16 and Ki-67 demonstrated high diagnostic sensitivity for the detection of CIN2+ lesions (95.5%), with a specificity of 71.2%. For both markers, the PPV was 78.8%, while the NPV reached 93.3%, indicating that negative test results have a significant ability to exclude CIN2+ lesions. These findings suggest that p16 and Ki-67 are reliable biomarkers for identifying women at increased risk of clinically significant cervical lesions.

Table 10 presents the expression patterns of p16 and Ki-67 among women with histologically confirmed CIN2+ lesions, comparing those with negative or low-grade cytological findings and those with high-grade cytological abnormalities.

A significantly higher proportion of women with CIN2+ lesions and low-grade cytology expressed p16 and Ki-67 than all other participants (91.9% vs. 100.0% (*p* = 0.249). Conversely, double negativity for both markers was considerably less frequent in the CIN2+ group (8.1% vs. 36.0%). These findings suggest that combined p16 and Ki-67 positivity may be a useful indicator for identifying clinically significant cervical lesions even among women presenting with low-grade cytological abnormalities.

Table 11 presents the results of the univariate logistic regression analysis.

The univariate logistic regression analysis demonstrated that age, parity, smoking, irregular participation in Pap smear screening, the presence of postcoital bleeding, high-grade cytological abnormalities, positive p16 and Ki-67 biomarker expressions, and HPV16 infection were significantly associated with an increased risk of CIN2+ lesions. Age was identified as a significant predictor of CIN2+ lesions (OR = 1.064, 95% CI: 1.025–1.105, *p* = 0.001), indicating that the odds of having a CIN2+ lesion increased by approximately 6.4% with each additional year of age. Higher parity was also associated with an increased risk of CIN2+ lesions (OR = 1.320, 95% CI: 1.041–1.575, *p* = 0.022), corresponding to an approximately 32% increase in risk with each additional childbirth. Smoking was significantly associated with the presence of CIN2+ lesions (OR = 1.989, 95% CI: 1.076–3.675, *p* = 0.028), indicating that smokers had approximately twice the odds of developing high-grade cervical lesions compared with non-smokers. Women who did not undergo Pap smear screening at the recommended intervals had nearly a threefold higher risk of CIN2+ lesions (OR = 2.836, 95% CI: 1.249–6.442, *p* = 0.013). In contrast, regular gynecological examinations alone were not significantly associated with the presence of CIN2+ lesions (*p* = 0.353). The presence of postcoital bleeding was identified as a significant clinical predictor of CIN2+ lesions (OR = 4.366, 95% CI: 1.513–12.600, *p* = 0.006). Women reporting this symptom had more than fourfold higher odds of harboring high-grade cervical lesions compared with those without postcoital bleeding. The cytological findings showed that low-grade cytological abnormalities (ASC-US, AGC-NOS, and LSIL), as well as the ASC-H/AGC-FN category, were not significantly associated with CIN2+ lesions. In contrast, high-grade cytological abnormalities (HSIL, >HSIL, AIS, and >AIS) were a strong predictor of CIN2+ lesions (OR = 19.500, 95% CI: 2.286–106.308, *p* = 0.007). Although the confidence interval was wide, likely reflecting the limited number of participants in some cytological categories, the observed association indicates a markedly increased risk of CIN2+ lesions among women with high-grade cytological abnormalities. TZ3 was not significantly associated with the presence of CIN2+ lesions. Compared with the Type I transformation zone, neither Type II (OR = 1.276, *p* = 0.636) nor Type III (OR = 1.740, *p* = 0.163) was associated with a significantly increased risk. Regarding colposcopic findings, Grade 1 abnormalities were associated with significantly lower odds of CIN2+ lesions compared with the reference category (Grade 0) (OR = 0.371, 95% CI: 0.159–0.868, *p* = 0.022). Grade 2 abnormalities showed a trend toward an increased risk (OR = 2.629, 95% CI: 0.965–7.159), although this association did not reach statistical significance (*p* = 0.059). The strongest association with CIN2+ lesions was observed for dual p16/Ki-67 biomarker positivity. Women with positive expression of both biomarkers had more than 50-fold higher odds of CIN2+ lesions than those with negative biomarker expression (OR = 51.882, 95% CI: 14.311–188.097, *p* < 0.001). Among the HPV genotypes evaluated, HPV16 infection was significantly associated with CIN2+ lesions (OR = 2.759, 95% CI: 1.311–5.807, *p* = 0.008), with HPV16-positive women exhibiting nearly threefold higher odds of developing high-grade cervical lesions. In contrast, HPV18 infection was not significantly associated with CIN2+ lesions (OR = 0.714, 95% CI: 0.300–1.699, *p* = 0.447). Overall, the results of the univariate logistic regression analysis indicate that dual p16/Ki-67 positivity, high-grade cytological abnormalities, HPV16 infection, and postcoital bleeding exhibited the greatest predictive value for the presence of CIN2+ lesions. These variables were therefore considered the most relevant candidates for inclusion in the multivariable logistic regression model to identify independent predictors of CIN2+ lesions (Table 12).

Abnormal cytological findings were the strongest independent predictor of CIN2+. After adjustment for smoking status, and HPV genotype, women with abnormal cytology had approximately 22-fold higher odds of histopathologically confirmed CIN2+ than those with normal cytological findings (OR = 21.8). The presence of HPV16 and/or HPV18 was associated with approximately twofold increased odds of CIN2+ (OR = 2.10) compared with other high-risk HPV genotypes. In the multivariable logistic regression analysis, current smoking was independently associated with lower odds of CIN2+ lesions (OR = 0.036, 95% CI: 0.003–0.425, *p* = 0.008), (Table 12).

After simultaneous adjustment for all variables in the model, age, positive Ki-67 biomarker expression, and the presence of postcoital bleeding remained independent predictors of CIN2+ lesions, whereas the association between HPV16 infection and CIN2+ lesions was no longer statistically significant. Age remained independently associated with the presence of CIN2+ lesions (OR = 1.061, 95% CI: 1.007–1.119, *p* = 0.027), indicating that the odds of CIN2+ lesions increased by approximately 6% with each additional year of age. The strongest independent predictor in the model was positive Ki-67 expression (OR = 75.692, 95% CI: 14.987–382.278, *p* < 0.001). Women with positive Ki-67 expression had approximately 76-fold higher odds of harboring CIN2+ lesions than those with negative Ki-67 expression, independent of age, HPV16 infection, or the presence of postcoital bleeding. This finding highlights the remarkable diagnostic and prognostic value of Ki-67 as a biomarker for identifying women at high risk of high-grade cervical lesions. The presence of postcoital bleeding also remained an independent risk factor for CIN2+ lesions (OR = 7.452, 95% CI: 1.313–42.310, *p* = 0.023). Women reporting postcoital bleeding had more than sevenfold higher odds of CIN2+ lesions than those without this symptom. Although HPV16 infection was a significant predictor in the univariate analysis, it did not remain statistically significant after adjustment for the other variables included in the model (OR = 1.870, 95% CI: 0.623–5.617, *p* = 0.264).

The results of the multivariable logistic regression analysis are presented in Table 13. 

The multivariable logistic regression analysis demonstrated that positive p16 expression and the presence of postcoital bleeding were independent predictors of CIN2+ lesions (Table 13). After simultaneous adjustment for age, HPV16 infection, postcoital bleeding, and p16 expression, p16 remained the strongest factor associated with the presence of CIN2+ lesions. Positive p16 expression was associated with approximately 55-fold higher odds of CIN2+ lesions compared with negative p16 expression (OR = 54.933, 95% CI: 13.170–229.139, *p* < 0.001). The presence of postcoital bleeding also remained significantly associated with CIN2+ lesions (OR = 4.912, 95% CI: 1.025–23.552, *p* = 0.047). Women reporting postcoital bleeding had approximately fivefold higher odds of harboring CIN2+ lesions than those without this symptom, independent of the other variables included in the model. Age did not retain statistical significance after adjustment for the other covariates (OR = 1.044, 95% CI: 0.993–1.097, *p* = 0.095), although a trend toward increased risk with advancing age was observed. Similarly, HPV16 infection did not reach statistical significance in the multivariable model (OR = 2.835, 95% CI: 0.974–8.250, *p* = 0.056), although it showed a tendency toward an increased risk of CIN2+ lesions (Table 14).

## 4. Discussion

In our study population of 125 HPV-positive women, histopathologically confirmed CIN2+ lesions were identified in 66 (52.8%). This high proportion of clinically significant lesions does not indicate the prevalence of CIN2+ lesions in the general female population; rather, it is a consequence of the selected study population, which consisted exclusively of HPV-positive women referred for further diagnostic evaluation. Nevertheless, this characteristic of the study cohort increases the clinical relevance of our findings. It reflects the population of women most commonly encountered in routine gynecological practice following a positive HPV test, in whom decisions regarding further diagnostic management must be made. The most important finding is that a substantial proportion of CIN2+ lesions occurred in women whose cytological findings did not indicate high-grade disease. Overall, 56.0% of women with histopathologically confirmed CIN2+ lesions had negative or low-grade cytological findings, including NILM, ASC-US, AGC-NOS, and LSIL. Notably, CIN2+ lesions were confirmed in 40.0% of women with NILM cytology. This finding directly supports the main hypothesis of our study that negative cytology in an HPV-positive woman should not be considered sufficient evidence to safely defer further diagnostic evaluation. A similar clinical dilemma has been described in the recent literature [32,33]. Among HPV-positive women with negative cytology, the greatest challenge is not the detection of HPV infection itself but rather the identification of those harboring biologically significant cervical lesions [32]. Therefore, the triage of HPV-positive women should be sensitive enough to detect clinically significant lesions while maintaining adequate specificity to avoid unnecessary colposcopies, biopsies, and excisional procedures. In a validation study of the ASCCP risk-based management guidelines in a Chinese population, Gui et al. demonstrated that discrepancies between recommended clinical management and subsequently detected CIN3+ lesions occurred predominantly among HPV-positive women with NILM cytology, particularly those infected with HPV16 and/or HPV18 [34]. Our findings are consistent with these observations and indicate that a negative cytological result should not automatically be equated with low risk in all HPV-positive women. In our cohort, 28.0% of women with negative cytology were positive for high-risk HPV (hrHPV), whereas previous studies have reported a prevalence of approximately 16.5% [35]. Furthermore, our results demonstrated that a considerable proportion of hrHPV-positive women with negative or low-grade cytological findings harbored clinically significant cervical lesions (CIN2+) that would otherwise have remained undetected and untreated. These findings raise concerns regarding the safety of postponing further diagnostic evaluation based solely on apparently normal cytological findings. False-negative cytology represented one of the most clinically relevant observations of the present study. Among women with histopathologically confirmed CIN2+ lesions, 21.2% had completely normal cytology, while an additional 34.3% exhibited cytological abnormalities of a lower grade than the final histopathological diagnosis. These findings further highlight the limited sensitivity of conventional cytology in the triage of hrHPV-positive women and are consistent with previous reports demonstrating the limitations of cytology-based triage. Piotrowski et al. reported that approximately 15% of hrHPV-positive women with initially negative cytology developed abnormal cytological findings during follow-up, raising further questions about the safety of relying exclusively on negative cytological results [31]. One particularly important finding of the present study was the progressive increase in false-negative cytological results with advancing age. Women older than 55 years exhibited the highest proportion of CIN2+ lesions despite having normal or low-grade cytological findings (40.0%). Reduced cellularity and atrophic epithelial changes associated with the postmenopausal period may contribute to the decreased sensitivity of cytological screening in older women. These findings are supported by previous studies evaluating the performance of cytology in hrHPV-positive postmenopausal women. Kiff et al. demonstrated that primary hrHPV testing may provide greater clinical benefit than co-testing in postmenopausal women because of the reduced sensitivity of cytology in a hypoestrogenic environment [27]. A further diagnostic challenge in this population is the limited performance of colposcopy. Kunkel et al. reported that more than 85% of women older than 65 years with persistent hrHPV infection and NILM cytology had unsatisfactory colposcopic examinations due to a type 3 transformation zone and a non-visible squamocolumnar junction, substantially reducing the diagnostic accuracy of colposcopy in this setting [36]. Our findings further confirm these limitations among hrHPV-positive women. The colposcopic findings of the present study were significantly associated with the final histopathological diagnosis. Grade 2 colposcopic abnormalities were more frequently observed in women with CIN2, CIN3, and invasive cervical cancer, whereas Grade 1 abnormalities predominated among women with cervicitis and CIN1. These findings support the important role of colposcopy in the triage of HPV-positive women and indicate that more severe colposcopic abnormalities are associated with an increased likelihood of clinically significant cervical disease. At the same time, our results highlight notable limitations of colposcopy. Unsatisfactory colposcopic examinations were observed in 43.2% of all participants and in 57.7% of women with invasive cervical cancer. Among women with CIN2+ lesions despite negative or low-grade cytology, an unsatisfactory colposcopic examination was the most common finding, occurring in 51.4% of cases. Thus, in the very subgroup in which cytology failed to indicate a high risk of disease, colposcopy was also frequently inadequate. This finding has important clinical implications. An unsatisfactory colposcopic examination should not be interpreted as a normal finding, particularly in HPV-positive women. Rather, it represents a diagnostic limitation because clinically significant lesions may be located within the endocervical canal and therefore remain inaccessible to direct visualization. In this context, a TZ3 deserves particular attention. Although TZ3 was not significantly associated with the final histopathological diagnosis in our study, it was most frequently observed among women with invasive cervical cancer. Moreover, women with a TZ3 were generally older, and positive p16 and Ki-67 expressions were frequently detected in this subgroup. Particular attention was paid to the type of transformation zone, as inadequate visualization of the squamocolumnar junction and a TZ3 may reduce the reliability of colposcopic assessment and hinder the detection of endocervically located lesions. In the univariate logistic regression analysis, the type of transformation zone was not significantly associated with the presence of CIN2+ lesions. Compared with the type 1 transformation zone, neither type 2 nor TZ3 was associated with a statistically significant increase in the risk of CIN2+. Regarding the colposcopic findings, a G1 colposcopic impression was associated with a lower likelihood of CIN2+ compared with the reference category (G0), whereas a grade 2 (G2) colposcopic impression showed a trend toward an increased risk, although the association did not reach statistical significance. Particular attention was paid to the type of transformation zone, as inadequate visualization of the squamocolumnar junction and a type 3 transformation zone may reduce the reliability of colposcopic assessment and hinder the detection of endocervically located lesions. In the univariate logistic regression analysis, the type of transformation zone was not significantly associated with the presence of CIN2+ lesions. Compared with the type 1 transformation zone, neither type 2 nor type 3 was associated with a statistically significant increase in the risk of CIN2+. Regarding the colposcopic findings, a G1 colposcopic impression was associated with a lower likelihood of CIN2+ compared with G0, whereas a G2 colposcopic impression showed a trend toward an increased risk, although the association did not reach statistical significance. Bruno et al. investigated women with persistent HPV infection, negative cytology, and a TZ3 and demonstrated that this clinical scenario is associated with a clinically relevant risk of CIN2+ lesions, indicating that negative cytology alone is insufficient to exclude significant cervical pathology [37]. Similarly, studies evaluating colposcopy in older HPV-positive women have shown that, with increasing age, the transformation zone progressively retracts into the endocervical canal, reducing the diagnostic accuracy of colposcopy and increasing the importance of additional triage biomarkers and risk stratification tools. Age was significantly associated with the histopathological diagnosis, and in the multivariable model including Ki-67, it remained an independent predictor of CIN2+ lesions. The likelihood of CIN2+ lesions increased with each additional year of age. This finding may be explained by the cumulative effect of persistent HPV infection, the reduced probability of spontaneous viral clearance in older women, and the progressive retraction of the transformation zone into the endocervical canal with advancing age. Smoking was associated with approximately twofold higher odds of CIN2+ lesions in the univariate analysis and was also significantly associated with the severity of histopathological findings. The highest proportion of smokers, particularly women smoking one pack of cigarettes per day, was observed among patients with cervical cancer. These findings are consistent with a large body of evidence identifying smoking as an important cofactor in cervical carcinogenesis. In the multivariable logistic regression analysis, current smoking was independently associated with lower odds of CIN2+ lesions. Recent studies integrating observational and genetic evidence have demonstrated that active smoking is associated with an increased risk of cervical cancer. Proposed biological mechanisms include local immunosuppression, impaired clearance of HPV infection, oxidative stress, chronic inflammation, and smoking-induced epigenetic alterations [38]. Earlier studies also detected nicotine and cotinine in cervical mucus, suggesting that tobacco smoke constituents may exert direct carcinogenic effects on the cervical epithelium [38]. Meta-analytic evidence further suggests that the interaction between smoking, HPV infection, and genetic polymorphisms involved in xenobiotic detoxification may further increase the risk of cervical cancer [39,40]. Prospective cohort data from Korea demonstrated that smoking was associated with increased cervical cancer mortality, whereas failure to participate in cervical screening was likewise associated with poorer clinical outcomes [40]. In addition, several studies have reported that smoking adversely affects the prognosis of locally advanced cervical cancer and that genetic polymorphisms of detoxification enzymes, such as *GSTP1*, may modify the association between smoking and cervical cancer risk [41,42]. Taken together, our findings further support the inclusion of smoking status as a clinically relevant factor in the risk assessment of HPV-positive women, particularly in cases in which cytological findings do not clearly indicate high-grade disease. Smoking was prevalent among women with CIN2+ lesions and low-grade cytological findings. Based on our results, smokers had approximately 3.2-fold higher odds of histopathologically confirmed CIN2+ than non-smokers. Roteli-Martins et al. demonstrated a strong association between cigarette smoking and high-grade CIN lesions, reporting a 6.6-fold increased risk of CIN2/3 among smokers [43]. Smoking-related immune dysregulation and chronic cervical inflammation may further contribute to the persistence of hrHPV infection. Recent studies have also shown that non-HPV16/18 high-risk genotypes, such as HPV52, HPV58, and HPV33, may be associated with clinically significant cervical lesions despite negative cytology [8]. These findings suggest that reliance solely on HPV16/18 genotyping may underestimate clinically relevant disease in certain patients. Among non-HPV16/18 infections in our study, the genotypes of particular oncogenic relevance in triage included HPV31, HPV53, and HPV58 [44]. Postcoital bleeding emerged as one of the most important clinical factors in the present study. It was strongly associated with the histopathological diagnosis, particularly with the presence of invasive cervical cancer, and remained an independent predictor of CIN2+ lesions in both multivariable models. In the model including Ki-67, women with postcoital bleeding had more than sevenfold higher odds of CIN2+ lesions, whereas in the p16-based model, the risk was approximately fivefold higher. This finding has direct clinical implications. In HPV-positive women, the presence of postcoital bleeding should not be overlooked, even when cytological findings do not indicate high-grade abnormalities [29], as this symptom may represent an underlying lesion requiring prompt diagnostic evaluation. Regular annual gynecological visits were not significantly associated with the histopathological diagnosis, whereas previous participation in Pap smear screening was significantly associated with outcomes in the univariate analysis. This finding indicates that merely attending gynecological examinations is insufficient if appropriate screening is not performed at recommended intervals and if abnormal findings are not managed according to established clinical algorithms. In settings where organized CC screening coverage remains suboptimal, information regarding previous screening participation and continuity of follow-up should be considered an essential component of the overall risk assessment of HPV-positive women. The value of the present study lies in assessing the biological potential of histopathologically confirmed lesions rather than in replacing the cytological p16/Ki-67 dual-stain test as a primary triage tool. Wright et al. [45] in the IMPACT study, demonstrated that p16/Ki-67 dual-stain cytology is a more effective triage strategy for HPV-positive women than conventional cytology and partial HPV16/18 genotyping–based approaches. Compared with conventional triage, dual-stain cytology demonstrated higher sensitivity for the detection of CIN2+ and CIN3+, improved risk stratification, and greater reassurance in women with negative test results, irrespective of HPV genotype. Our findings do not directly demonstrate the clinical utility of cytological dual-stain testing in primary triage; however, they provide strong evidence supporting the biological relevance of these biomarkers in histopathologically confirmed lesions. This distinction has important practical implications. In healthcare systems where liquid-based cytology and validated p16/Ki-67 dual-stain cytology are available, these biomarkers may serve as a non-invasive triage tool before referral for colposcopy. In settings where conventional cytology is used, as in the present study, p16 and Ki-67 are primarily valuable for confirming the biological potential of histopathologically verified lesions. Nevertheless, their strong association with CIN2+ lesions supports the rationale for incorporating p16/Ki-67 dual-stain cytology into primary triage algorithms where the technology is available and validated. At 95%, the sensitivity of these biomarkers was high, which is comparable to values reported in the literature, where a sensitivity of approximately 92% has been described [20].

### 4.1. Strengths of the Study

The value of the present study lies in assessing the biological potential of histopathologically confirmed lesions rather than in replacing the cytological p16/Ki-67 dual-stain test as a primary triage tool. The use of multiple complementary diagnostic modalities, including extended HPV genotyping, colposcopy, histopathology, and immunohistochemical assessment of p16 and Ki-67, provided a comprehensive evaluation of cervical disease and improved the robustness of the findings. Paying particular attention to women with negative or low-grade cytology represents an additional strength, as this subgroup is clinically challenging and highly relevant for triage decision-making. Furthermore, the inclusion of socio-epidemiological factors, such as smoking status and screening behavior, adds a broader clinical context to the analysis.

The study was conducted under routine clinical conditions, thereby enhancing its real-world applicability. Unlike studies based on theoretically selected participants from a population-based model, this investigation included women encountered in everyday clinical practice who tested positive for HPV and were referred for further diagnostic evaluation. Therefore, the findings may contribute to optimizing the triage of HPV-positive women within the local healthcare system. All participants were enrolled as part of the standard diagnostic workup following a positive HPV test. The study population was initially analyzed as a single cohort of HPV-positive women. For subsequent analyses, however, participants were stratified according to cytological findings, HPV genotype, colposcopic findings, transformation zone type, histopathological diagnosis, p16 and Ki-67 expression, age, smoking status, and other relevant clinical and demographic characteristics.

### 4.2. Limitations of the Study

However, several limitations should be acknowledged. First, the study was conducted at a single tertiary care center, which may limit the generalizability of the results to broader populations. Second, the relatively small sample size and the inclusion of women referred for colposcopic and histopathological evaluation may have introduced selection bias, resulting in a higher proportion of CIN2+ lesions than would be expected in an unselected screening population. Therefore, the observed frequency of CIN2+ lesions should not be interpreted as the prevalence of disease among all HPV-positive women, and caution is warranted when generalizing these findings to broader screening populations. Third, p16/Ki-67 dual-stain testing was performed on histopathological tissue samples rather than directly on cytological specimens, which limits direct comparison with current clinical screening algorithms where dual-stain testing is typically applied to cytology. Additionally, the prospective hospital-based observational study does not allow for assessment of longitudinal outcomes or progression of lesions over time. Finally, potential selection bias may exist, as only women with positive hrHPV results referred for further evaluation were included in the study.

## 5. Conclusions

Our study demonstrates that a substantial proportion of hrHPV-positive women with negative or low-grade cytology harbor clinically significant CIN2+ lesions that may remain undetected if management relies solely on cytology. The findings underscore the limitations of conventional cytology and support a transition toward risk-based triage strategies integrating HPV genotyping, dual-stain biomarkers, and relevant clinical risk factors. Although p16/Ki-67 dual staining was evaluated on histological specimens, the observed diagnostic performance provides proof-of-concept for its implementation on cytological samples as a non-invasive triage tool. Such an integrated approach could improve individualized patient management, optimize colposcopy referrals, reduce unnecessary invasive procedures, and enhance healthcare resource utilization. These findings are particularly relevant in the era of primary HPV screening and may contribute to more effective cervical cancer prevention strategies aligned with global elimination goals.

## Figures and Tables

**Table 1:** Baseline characteristics of study participants.

Characteristics		Mean Value ± SD/*n* (%)
Age		46.51 ± 13.76
	30–35	24 (19.2)
	36–45	39 (31.2)
	46–55	38 (30.4)
	56–65	24 (19.2)
Parity	none	23 (18.4)
	1 child	20 (16.0)
	2 children	30 (24.0)
	3 children	27 (21.6)
	≥4 children	25 (20.0)
Regular annual gynecological examinations	No	86 (68.8)
	yes	39 (31.2)
Pap test each every 3 years	yes	88 (70.4)
	No	37 (29.6)
Smocking status	No	45 (36.0)
	One pack a day	70 (56.0)
	More than one pack a day	10 (8.0)
Family history	Negative	91 (72.8)
	Positive	34 (27.2)
Postcoital bleeding	No	101 (80.8)
	Yes	24 (19.2)

**Table 2:** Clinical, cytological, and histopathological characteristics of participants.

Identification	HPV Type	N %
HPV type	16/18	96 (76.8)
	Other types	29 (23.2)
Pap test	NILM	35 (28.0)
	ASC-US, AGC-NOS, LSIL	46 (36.9)
	ASC-H, AGC-FN	30 (24.0)
	HSIL, >HSIL, AIS, >AIS	14 (11.2)
Colposcopic examination	G0 unsatisfactory	54 (43.2)
	G1	41 (32.8)
	G2	30 (24.0)
Transformation zone type	TZ I	54 (43.2)
	TZ II	21 (16.8)
	TZ III	50 (40.0)
Histopathological examination	Cervicitis	38 (30.4)
	CIN 1	21 (16.8)
	CIN 2	15 (12.0)
	CIN 3	25 (20.0)
	Carcinoma	26 (20.8)
p16	Negative	45 (36.0)
	Positive	80 (64.0)
Ki-67	Negative	45 (36.0)
	Positive	80 (64.0)

**Table 3:** HPV types in study population.

HPV Type	Non-CIN2+	CIN2+	*p*
31	17 (11.9)	17 (13.6)	0.767
33	3 (5.1)	2 (3.0)	0.558
35	1 (1.7)	1 (1.5)	0.936
45	0 (0.0)	2 (3.0)	0.498
51	7 (5.6)	0 (0.0)	0.004
52	2 (3.4)	3 (4.5)	0.742
53	5 (8.5)	9 (13.6)	0.361
56	4 (3.2)	0 (0.0)	0.047
58	4 (6.8)	5 (7.6)	0.864
66	0 (0.0)	2 (3.0)	0.498
68	2 (3.4)	1 (1.5)	0.602
69	0 (0.0)	1 (1.5)	1.000

**Table 4:** Association between cytological findings and CIN2+ lesions.

Cytological Findings	Non-CIN2+	CIN2+	*p*
NILM	21 (35.6)	14 (21.2)	
ASCUS, AGCNOS, LSIL	23 (39.0)	23 (34.8)	
ASCH, AGC FN	14 (23.7)	12 (24.2)	
HSIL, IK, AK	1 (1.7)	13 (19.7)	0.009

**Table 5:** Age distribution of women with CIN2+ lesions and low-grade cytology (NILM, ASCUS, AGCNOS, LSIL) (*n* = 37).

Age Groups	*n* (%) *n* = 37	Of All the Women in the Population
30–35	3 (8.1)	3/24 (12.5)
36–45	12 (32.4)	12/39 (30.7)
46–55	13 (35.1)	13/38 (34.2)
56–65	9 (24.3)	9/24 (37.5)

**Table 6:** HPV infection characteristics in women with CIN2+ lesions and low-grade cytology (NILM and ASC-US/AGC-NOS/LSIL).

CIN2+ Lesions	Characteristics	N 37%
Non-HPV 16/18	Positive	8 (21.6)
HPV 16/18	Positive	29 (78.4)
Number of HR-HPV infections	Monotype	30 (81.1)
	Polytype	7 (18.9)

**Table 7:** Colposcopic findings in women with CIN2+ lesions according to cytological findings.

Colposcopic Findings	NILM and ASC-US/AGC-NOS/LSIL *n* = 37	All Other ASCH, AGC-FN HSIL, >HSIL, AIS > AIS	*p*
Unsatisfactory	19 (51.4)	11 (37.9)	
G1	8 (21.6)	5 (17.2)	
G2	10 (27.0)	13 (44.8)	0.319

**Table 8:** Association between p16 and Ki-67 expressions and histopathological findings.

		Cervicitis	CIN 1	CIN2	CIN3	Carcinoma	*p*
p16	Negative	31 (81.6)	11 (52.4)	2 (13.3)	0 (0.0)	1 (3.8)	
	Positive	7 (18.4)	10 (47.6)	13 (86.7)	25 (100.0)	25 (96.2)	<0.001
Ki-67	Negative	33 (86.8)	9 (42.9)	1 (3.8)	1 (3.8)	1 (3.8)	<0.001
	Positive	5 (13.2)	12 (57.1)	14 (93.3)	24 (96.0)	26 (96.2)	<0.001

**Table 9:** Sensitivity, specificity, NPV, and PPV for p16 and Ki-67 markers.

Biomarkers	Characteristics	Non-CIN2+	CIN2+	*p*
p16	Negative	42 (71.2)	3 (4.5)	
	Positive	17 (28.8)	63 (95.5)	<0.001
Ki-67	Negative	42 (71.2)	3 (4.5)	
	Positive	17 (28.8)	63 (95.5)	<0.001

**Table 10:** p16 and Ki-67 expressions in women with negative or low-grade cytological findings according to histopathological diagnosis (CIN2+ versus cervicitis).

		NILM and ASC-US/AGC-NOS/LSIL *n* = 37	All Other ASCH, AGC-FN HSIL, >HSIL, AIS > AIS	*p* *
p16	Negative	3 (8.1)	0 (0.0)	
	Positive	34 (91.9)	29 (100.0)	0.249
Ki-67	Negative	3 (8.1)	0 (0.0)	
	Positive	34 (91.9)	29 (100.0)	0.249

* Fisher’s test.

**Table 11:** Univariate logistic regression analysis of risk factors for CIN2+.

Variables		OR	95% CI	*p*
Age		1.064	1.025–1.105	0.001
Parity		1.320	1.041–1.575	0.022
Smoking		1.989	1.076–3.675	0.028
Regular gynecological examinations		1.437	0.669–3.0900	0.353
Pap smear screening at the recommended intervals		2.836	1.249–6.442	0.013
Positive family history		1.008	0.458–2.219	0.985
Postcoital bleeding		4.366	1.513–12.600	0.006
Cytological finding	NILM	/	/	/
	ASCUS, AGC-NOS, LSIL	1.5600	0.616–3.651	0.372
	ASCH, AGC-FN	1.714	0.640–4.594	0.284
	HSIL, >HSIL, AIS, >AIS	19.500	2.286–106.308	0.007
Transformation zone type	TRZ I	/	/	/
	TRZ II	1.276	0.465–3.5002	0.636
	TRZ III	1.740	0.799–3.791	0.163
Colposcopic finding	G0 ref	/	/	/
	G1	0.371	0.159–0.868	0.022
	G2	2.629	0.965–7.159	0.059
p16		51.882	14.311–188.097	<0.001
Ki-67		51.882	14.311–188.097	<0.001
HPV16		2.759	1.311–5.807	0.008
HPV18		0.714	0.300–1.699	0.447

OR—odds ratio; CI—confidence interval.

**Table 12:** Multivariable logistic regression analysis of risk factors associated with CIN2+ lesions.

Variables	OR	95% CI	*p*
Current smoker	0.036	0.003–0.425	0.008
HPV16/HPV18	2.10	1.09–4.03	0.026
Abnormal cytology	2.78	6.23–76.14	<0.001

OR—odds ratio; CI—confidence interval.

**Table 13:** Multivariable logistic regression analysis of risk factors associated with CIN2+ lesions.

Variables	OR	95% CI	*p*
Age	1.061	1.007–1.119	0.027
Postcoital bleeding	7.452	1.313–42.310	0.023
Ki-67	75.692	14.987–382.278	<0.001
HPV16	1.870	0.623–5.617	0.264

OR—odds ratio; CI—confidence interval.

**Table 14:** Multivariable logistic regression analysis of risk factors associated with CIN2+ lesions.

Variables	OR	95% CI	*p*
Age	1.044	0.993–1.097	0.095
Postcoital bleeding	4.912	1.025–23.552	0.047
p16	54.933	13.170–229.139	<0.001
HPV16	2.835	0.623–5.617	0.056

OR—odds ratio; CI—confidence interval.

## Data Availability

The original contributions presented in this study are included in the article. Further inquiries can be directed to the corresponding author.

## Abbreviations

HPV	*Human papillomavirus*
hrHPV	HrHPV—High-risk HPV
ACS	The American Cancer Society
AGC	atypical glandular cells
ASCCP	American Society for Colposcopy and Cervical Pathology
ASC-H	Atypical squamous cells cannot exclude high-grade squamous intraepithelial lesion
ASC-US	atypical squamous cells of undetermined significance
CAP	The College of American Pathologists
CIN	cervical intraepithelial neoplasia
HSIL	high-grade squamous intraepithelial lesion
LSIL	low-grade squamous intraepithelial lesion
TBS	The Bethesda system
